# Impact of Eco-Friendly Plaster Using Epoxy Resin and Epoxy Hardener on Mechanical Properties under Compression and Tension

**DOI:** 10.3390/polym16040548

**Published:** 2024-02-18

**Authors:** Mohammed A. Albadrani, Ahmed D. Almutairi

**Affiliations:** 1 Department of Mechanical Engineering, College of Engineering, Qassim University, Buraydah 51452, Saudi Arabia; 2 Department of Civil Engineering, College of Engineering, Qassim University, Buraydah 51452, Saudi Arabia; ah.almutairi@qu.edu.sa

**Keywords:** compression test, eco-friendly, modeling, sustainability, tensile test, mechanical properties, epoxy

## Abstract

BISCO plaster (BRP) is an environmentally friendly material with high mechanical properties and is considered a great elective to conventional materials such as gypsum and cement. Our investigation seeks to examine BISCO plaster (BRP) and a mixture of resin and hardener in three proportions (30%, 45%, and 60%) to achieve our ultimate goal, which is to preserve the environment and achieve the vision of the Kingdom of Saudi Arabia 2030 to reach zero carbon emissions by 2060? Emissions tests were performed, and although the CO_2_ level was zero, they emitted SO_2_ sulfur dioxide and NO_2_ nitrogen dioxide, and 60% was the lowest emission rate. We also used ANSYS 2023 R1 software to compare them with their mechanical properties resulting from tensile and compression testing. In this study, we looked closely at the mechanical characteristics of different materials designed for wall coverings, with particular emphasis on their environmental sustainability. We carried out experiments to gauge the tensile and compressive stress on samples with varying mixing ratios. Our main objective was on crucial mechanical properties such as the modulus of elasticity, ultimate tensile strength, yield strength, yield strain, modulus of resilience, and ductility. Through meticulous scrutiny, we determined that the amalgamation of these mechanical attributes at the 30% mixing ratio provides an optimal combination for attaining structural integrity, adaptability, and resilience in wall coverings. Significantly, this ratio also underscores a commitment to environmentally conscious material selection. Our study offers important new insights into the selection of wall covering materials by providing a detailed understanding of their mechanical behavior under various stress conditions. It aligns with the increasing significance of environmental responsibility in contemporary design and construction. By emphasizing the 30% mixing ratio, our findings establish a foundation for informed decision making, promoting the utilization of sustainable materials that achieve a balance between strength, flexibility, and longevity. This ensures optimal performance in practical applications while simultaneously minimizing the environmental impact.

## 1. Introduction

### Background

Green building (commonly referred to as environmentally friendly or sustainable buildings) is an architecture and procedure that is environmentally conscious and utilizes resources effectively through the life cycle of a building, from sitting to design, construction, operation, maintenance, renovation, and demolition [1]. Sustainable structures necessitate the use of improved performance materials that have good mechanical behavior. Nowadays, eco-friendly plaster is the focus due to its significantly lower carbon footprint compared to synthetic alternatives. Its production process requires less energy and generates fewer greenhouse gas emissions, contributing to a more sustainable building industry [2]. One of the most important aspects of these new materials is their prolonged life or durability over time, which reduces the need for restorations and, as a result, the environmental impact of disposal [3]. One of the biggest durability issues with external plasters is the impact of atmospheric agents on the building’s surface, particularly the many kinds of water that affect the porous nature of the materials. To ensure sustainability and healthy living, eco-friendly materials are increasingly being used in the construction industry. Plasters have strong durability but are fragile in mechanical performance; hence, strengthening these plasters has been found to be useful at the post-tensioning stage. In this regard, more recently, improvements were presented to improve the performance of the plaster. Lime–Pozzolan Plasters with Self-Cleaning Properties for Bioconstruction were developed to evaluate the effectiveness and endurance of novel mortars in order to properly use them and avoid irreversible damage over time. In this study, lime–metakaolin and hydraulic lime–metakaolin-based mortars, as well as mortars containing nano-TiO_2_ and perlite, were tested. The obtained results showed degradation effects in the mortar samples due to ageing following every test and demonstrated that the mortars with perlite and nano-TiO_2_ are the best-performing ones, in addition to terms of durability and energy, making them appropriate for applications in the environmentally friendly construction sector [4]. Physical parameters like setting time, temperature rise, and density were examined to evaluate the efficacy of this environmentally friendly construction plaster made with Prosopis juliflora fibers. In addition, the Prosopis juliflora fibers were treated with a variety of chemical treatments. The addition of the fibers significantly improved the toughness of the composites [5].

Research and development in the realm of epoxy polymers was a highly topical subject during the 1970s and 1980s, a time when the fundamental knowledge surrounding thermosetting polymers was being established. This period marked a significant understanding of the various chemistries involved in the synthesis of epoxy networks, as well as the description of network formation through different levels of complexity. Furthermore, transitions that occurred during network formation, such as gelation and verification, were both described and predicted. Transformation diagrams were also developed to provide a rationalized approach to cure cycles, and the relationship between structure and properties was accurately established based on model systems. Simultaneously, the industry was also making strides in the development of new formulations and processing techniques for the diverse applications of epoxy polymers, including adhesives, coatings, and composites.

These applications expanded into various sectors, such as the building, electronics, sports goods, automobile, and aircraft industries. However, what was once considered an established field by the end of the 1990s experienced a strong revitalization at the start of the new century for several reasons. Firstly, the advent of the “nano” prefix, which has permeated various fields, has also brought about significant changes in the epoxy domain. Formulations containing nanoclays, polyhedral oligomeric silsesquioxanes (POSS), block copolymers (BCP), carbon nanotubes (CNT), and other such components are continuously being developed, tested, and commercialized.

Epoxy polymers are increasingly finding important applications in expanding fields such as the manufacturing of windmill blades for the conversion of eolic energy into electricity, as well as foams for electronic applications. New formulations based on epoxy acrylates have emerged as high-performance coatings, and thermoplastic epoxies are now competing with conventional thermoplastic polymers in many applications. These commercially available formulations can be processed like thermoplastics and cured like thermosets. Additionally, new processing techniques that provide high cure rates are now accessible. The concept of “green chemistry” has also made its way into the epoxy field, with different monomers derived from a variety of natural products now available, and their inclusion in commercial formulations is expected to increase. Moreover, epoxy polymers also contribute to the development of advanced functional materials, as they have the potential to exhibit self-healing properties, shape memory effects, and transparent–opaque transitions, among other advanced characteristics [6].

Epoxy resin, a highly significant and well-received category of thermosetting polymers, is deeply ingrained in numerous domains, encompassing our everyday existence as well as the industrial sector. By virtue of its exceptional mechanical robustness and dimensional and thermal steadiness, along with its resistance to deformation, chemicals, and electrical insulation, epoxy resin has achieved extensive application in fields such as construction, adhesives, electronic and electrical apparatus, coatings, composites, and more. Nevertheless, the insolubility and infusibility inherent in these irreversible covalent networks impede the possibility of recycling and reprocessing epoxy resin [7].

The IUPAC nomenclature for an epoxide functional group is an oxirane. Epoxy resins can undergo cross-linking either with themselves through catalytic homopolymerization or with a diverse array of co-reactants such as polyfunctional amines, acids (as well as acid anhydrides), phenols, alcohols, and thiols (also known as mercaptans). These co-reactants are commonly denoted as hardeners or curatives, and the cross-linking reaction is typically referred to as curing. The reaction between polyepoxides and either themselves or polyfunctional hardeners leads to the formation of a thermosetting polymer, often exhibiting favorable mechanical properties and high thermal and chemical resistance. Epoxy finds extensive utility in various applications, including metal coatings and composites [8].

We currently have concerns about the environmental impacts of common industrial products, such as volatile organic compounds and hazardous chemicals that threaten human health and the environment, so researchers are looking for sustainable alternatives that can replace hazardous materials without affecting performance. It is important for its ability to enhance mechanical properties in industrial manufacturing. When mixing different materials, such as concrete, steel, and plaster, the resulting mix shows strength, durability, and corrosion resistance. This makes epoxy an attractive choice for companies looking to improve the performance of their products. The aim of this study is to study the effect of different epoxy formulations in plastering mixtures on the mechanical properties of the resulting chemicals by conducting experiments and analyzing the data [9].

The utilization of epoxy resin in polymer concrete offers numerous benefits, enhancing the material’s performance and contributing to environmental sustainability. This report examines the advantages of epoxy resin in polymer concrete, including improved strength, chemical resistance, and adhesion. Furthermore, it explores the eco-friendly aspects, such as enhanced durability, reduced maintenance needs, resistance to chemicals and corrosion, lower carbon footprint, reduced water usage, recycling potential, versatility in design, and reduced energy consumption during manufacturing.

- Enhanced Strength

The combination of epoxy resin and concrete aggregates results in a composite material with higher tensile and flexural strength. The epoxy resin acts as a binder, reinforcing the concrete and enhancing its overall strength.

- Chemical Resistance

Epoxy resin provides polymer concrete with increased resistance to chemicals, making it suitable for environments where traditional concrete might deteriorate. This chemical resistance minimizes the risk of damage and extends the lifespan of structures in corrosive environments.

- Adhesion

Epoxy resin improves the adhesion between the aggregates in polymer concrete, leading to a more cohesive and durable material. This enhanced adhesion strengthens the structure and reduces the likelihood of delamination or cracking.

- Enhanced Durability and Longevity

The incorporation of epoxy resins improves the durability and longevity of polymeric concrete. Structures built with epoxy-enhanced polymer concrete require fewer frequent repairs or replacements, reducing the overall consumption of resources and energy associated with construction activities.

- Reduced Maintenance Needs

Epoxy-enhanced polymer concrete typically requires less maintenance compared to traditional concrete. The reduced maintenance needs result in lower resource consumption and less waste generated from repair activities, contributing to a more sustainable construction industry.

- Resistance to Chemicals and Corrosion

The use of epoxy resin in polymer concrete imparts resistance to chemicals and corrosion. Structures with enhanced chemical resistance require fewer harsh chemicals for maintenance, reducing environmental contamination risks from chemical spills or leaching.

- Lower Carbon Footprint

Epoxy resins can contribute to reducing the carbon footprint of construction materials. Some epoxy resins can be derived from renewable sources or bio-based materials, potentially lowering the environmental impact associated with the production of petrochemical-based resins. By adopting epoxy-enhanced polymer concrete, the construction industry can move toward more sustainable practices.

- Reduced Water Usage

Epoxy-enhanced polymer concrete exhibits lower water absorption compared to traditional concrete. Structures with low water absorption are less prone to water damage, reducing the need for water-intensive repair and maintenance activities. This reduction in water usage contributes to water conservation efforts.

- Recycling Potential

Certain epoxy resins and polymer concrete formulations can be recycled. Recycling materials at the end of their life cycle reduces the demand for new raw materials and minimizes waste sent to landfills. The recycling potential of epoxy-enhanced polymer concrete aligns with the principles of a circular economy and promotes resource efficiency.

- Versatility in Design

Epoxy resins provide versatility in the design and application of polymer concrete. The ability to design structures with specific performance characteristics allows for more efficient use of materials and resources. This versatility enhances the overall sustainability of construction projects.

- Reduced Energy Consumption

Epoxy-enhanced polymer concrete may require lower curing temperatures compared to traditional concrete. Lower curing temperatures contribute to energy savings during the manufacturing process, reducing the overall carbon footprint associated with concrete production. This energy efficiency aligns with the goal of reducing greenhouse gas emissions in the construction industry.

Incorporating epoxy resin in polymer concrete offers significant advantages, including enhanced strength, chemical resistance, and improved adhesion. Additionally, the use of epoxy resins in polymer concrete promotes environmental sustainability through benefits such as enhanced durability and longevity, reduced maintenance needs, resistance to chemicals and corrosion, lower carbon footprint, reduced water usage, recycling potential, versatility in design, and reduced energy consumption during manufacturing. By leveraging these benefits, the construction industry can move toward more sustainable practices.

The use of epoxy resins in polymeric concrete not only improves the performance of the material but also has the potential to contribute to more sustainable and environmentally friendly construction practices. As the construction industry continues to prioritize eco-friendly solutions, the adoption of durable and efficient materials like epoxy-enhanced polymer concrete can play a significant role in reducing the environmental impact of infrastructure projects [10].

The risks of epoxy resin are due to the hazardous compounds used to cure it. Amine hardeners are one type that have an unpleasant odor and are used in homes. They cure at room temperature. Polymerization of epoxy resin releases heat and volatile substances, increasing toxicity. The main danger of epoxy resin is severe skin lesions. Direct contact with the liquid can cause skin irritations and allergic reactions. Dermatitis and respiratory tract irritation can also occur. To avoid these effects, gloves and a respirator with charcoal filters should be used.

To protect your breath when working with epoxy, it is necessary to use a respirator or half mask with organic vapor filters. These filters are also suitable for use with alcoholic inks. The majority of vapors are released when the components are mixed and when working with a burner. After pouring, the solid resin becomes harmless within 24–48 h. It is important to work in a well-ventilated room to minimize exposure to harmful substances during mixing and polymerization. When working with large quantities of resin, it is recommended to wear special safety glasses to protect the eyes. Avoid touching the face while working to prevent irritation and inflammation from resin contact with mucosa. To protect the skin, gloves and long-sleeved clothing should be worn from the time the resin packs are opened until the work area is cleaned [11].

Many recently were presented to study the characteristics of eco-friendly plaster, and the behavior effect of Epoxy Resin and Epoxy Hardener.

An eco-friendly composite gypsum binder with various mineral admixtures has been developed. It evaluated the impact of mineral additives on the mechanical properties of the composite gypsum binder at increasing ratios. This research also concentrated on the creation of a composite gypsum binder by employing a stepwise optimization technique to identify the appropriate dosage of each mineral admixture to achieve maximum mechanical strength. When compared to plain gypsum plaster, the synthesized composite gypsum binder with mineral admixtures demonstrated improved mechanical strength and decreased porosity [12].

Another investigation generated lightweight gypsum using hemihydrate phosphogypsum-based cementitious substance as the matrix component and a porous structure light-weight aggregate (LWA) as the lightweight component. The results reveal that lightweight gypsum with bulk densities varying from 300 kg/m^3^ to 1500 kg/m^3^ may be obtained. In terms of heat conductivity and sound absorption, lightweight gypsum with pottery sand has the smallest decrease while EPS particles have the greatest decrease [13].

Rubber waste was mixed with plaster-based composite to provide an alternative building material. The primary purpose of this study was to look at the efficacy of employing shredded rubber waste as aggregates in plaster mortar to improve its insulating capability. Mixing dune sand, plaster, rubber particles, and water yielded this composite. Rubber aggregates were used in mixes as a partial replacement for some sand by volume. The amount of rubber in the mix is used to analyze and compare unit weight, capillary absorption of water, mechanical, and thermal qualities. The results demonstrated that the addition of rubber changes the characteristics of the mortar. Despite the fact that the mechanical strength fell as the rubber content increased, it should be noted that rubber particles could greatly lower the weight of the material, reduce the rate of water absorption, and improve the insulating aspect of the composite. The study concluded that recycling rubber waste can provide an eco-friendly replacement material [14].

The influence of innovative pressure-induced plaster behavior on mechanical characteristics was examined. The researched plaster was an eco-friendly carbon-free BSCO plaster. A variety of experimental procedures were applied, including compression testing with the ANSYS 2023 R1 program for validation. The findings indicated that eco-friendly Bilateral Specialised Company (BSCO) Hafar Albatin, KSA plasters could have mechanical qualities equal to regular plasters. Furthermore, eco-friendly and carbon-free plasters are proposed to be a feasible alternative to traditional plasters in a variety of applications. Researchers and construction engineers were advised to replace traditional stucco with an environmentally friendly substitute with superior mechanical properties [15].

The results of an investigation into the thermal and mechanical characteristics of an eco-insulating material consisting of plaster and date palm fibers have been reported. Many aspects (physical, mechanical, and microstructure characteristics) were investigated experimentally to evaluate these materials. For mechanical, thermal, and physical characterizations, several samples of bio-composites of plaster configurations with short lengths (20 mm) and eight-weight ratios (0.5–4%) of palm fibers were created. According to the findings, plaster composites reinforced with date palm fibers can be classified as eco-friendly and thermally insulating building materials [16].

Epoxy resins are oligomers that possess epoxy groups and have the capability to create cross-linked polymers when exposed to hardeners, such as polyamines. These resins are available in both liquid and solid forms. While they are initially thermoplastic, the addition of various hardeners transforms them into non-melting polymers. By incorporating different additives like hardeners, fillers, plasticizers, thinners, etc., a wide range of materials with diverse properties based on epoxy resins can be obtained. Epoxy resins exhibit resistance to halogens, acids, and alkalis, and they are known for their exceptional mechanical strength, water resistance, high electrical strength, and strong adhesion to polar compounds, metals, porcelain, mica, and other substances. A notable advantage of epoxy resins is their minimal shrinkage during the process of solid-state transition. The primary distinguishing characteristic of epoxy resins is their ability, under specific conditions, to undergo a transformation into polymers with a mesh structure, rendering them suitable for the fabrication of various plastic materials, including compounds, adhesives, enamels, binders for laminated plastics, and sealants, among others. The formation of these polymer meshes is either a result of chemical reactions between the epoxy groups of the resin and a polyfunctional substance, or it occurs through the polymerization of epoxy groups facilitated by catalytically active compounds. In such instances, the polyfunctional substances are referred to as hardeners (or crosslinking agents), while the catalytically active substances are known as catalysts of curing (or catalytically active hardeners) [17].

Epoxy resins are commonly categorized into three distinct types: bisphenol-A, bisphenol-F, and novolac. Bisphenol-A is produced through the reaction of phenols with acetone, while the remaining types are formulated by the reaction of phenols with formaldehyde. The synthesis of novolac necessitates the use of excess phenol. Subsequently, these resins undergo a curing process by reacting with epichlorohydrin. Novolac epoxy resins are essentially modified versions of bisphenol-F resins, achieved by reacting excess phenol and formaldehyde. Amongst these resins, bisphenol-A is the most frequently utilized in coating systems due to its superior adhesion, chemical resistance, and wear resistance. It is crucial to acknowledge that, in addition to the base resins employed, the solidifier components, which consist of primary, secondary, or tertiary amine compounds, also exert a significant influence on the properties of the cured system [18].

Epoxy resins are suitable for reinforcing fibers due to their strong adhesion, low shrinkage, dimensional stability, and various desirable properties. Epoxy composites are created by aligning fibers in a resin matrix using various fabrication processes. Glass, boron, graphite, and polyaramides are commonly used fiber materials. The properties of the laminate are influenced by the orientation of the fibers. The resin–fiber interface is a critical region in composites. Epoxy resins are well suited for composite applications due to their adhesive properties. The main market for epoxy composites is corrosion-resistant equipment. Other markets include automotive, aerospace, sports/recreation, construction, and marine. Epoxy composites are used where their strength and resistance properties are advantageous [19].

Polymeric composite materials (PCM) can increase the durability of buildings and constructions. Epoxy resins are commonly used as binders in PCs due to their physical and mechanical characteristics. They have good adhesion, a variety of types, and are resistant to water and chemicals. Epoxy-based composites have high dielectric properties and good adhesion to various materials. Curing epoxy resins creates a cross-linked structure, allowing them to be converted into a thermosetting state [20].

Epoxy is used in various types of plaster for different purposes. The refractory epoxy high-polymer easy plaster [9] and the epoxy resin plaster for building jointing [21] both utilize epoxy resin as a key component. These plasters offer advantages such as excellent binding strength, impermeability, and fire resistance. Additionally, the epoxy resin plaster for building jointing is low in cost, quick to cure, and can be used in wet environments. Another application of epoxy in plaster is seen in the cationic ring-opening polymerization method for curing epoxy monomers in the restoration field [22]. This method uses a redox system to create a suitable consolidate for lime plaster, improving its mechanical strength and surface properties. Furthermore, a non-toxic epoxy plaster, especially for corrosion prevention, water resistance, and reinforcement [23], and a special silicone rubber/epoxy b-component plaster for a double-layer cold contraction tube [24] both utilize epoxy resin to enhance the performance of the plaster, such as improving corrosion resistance and ensuring high-grade waterproof sealing.

Different types and amounts of hardeners can change the properties of the composites. The nature of the hardener also affects the density of the spatial mesh in epoxy compositions. Polymers can be modified to improve the properties of composites [25].

The properties of polymers are affected by various factors, such as molecular weight “The properties of an epoxy resin are defined by weight per epoxide (WPE) that is the grams of resin per functional group: e.g., WPE 300 means one epoxide group per 300 g resin (14.3% in the molecule). Also, an epoxy resin is usually characterized by the weight per hydroxyl (WPH), which is the grams of resin per hydroxyl group in the structure: e.g., WPH 150 indicates the presence of a hydroxyl in 150 g resin (11.3% film-forming material composition)” [26], crosslinking, branching, segmental motion, morphology, and external conditions. The structure of side-chain substituents on the polymer backbone is an important factor that impacts polymer functionality. The type of side-chain substituent also has a significant effect on mechanical properties. Increasing the amount of highly polar side chains tends to increase tensile strength. The effect of side group structure on the compressive strength of biodegradable polyphosphazene-based polymers was investigated. The results showed that the nature and ratio of pendent groups attached to the polymer backbone play a role in determining mechanical properties [27].

A self-assembly approach based on coordination bonding and electrostatic interactions was used to build copper organophosphate nanosheets evenly on the surface of graphene oxide (GO). This study presented a novel interfacial approach for creating functional nanosheets in polymers with good interface compatibility and great flame-retardant efficacy [28].

A 9,10-dihydro-9-oxa-10-phosphaphenanthrene-10-oxide (DOPO)-derived flame retardant (PAHDOPO) was prepared by a neutralization reaction between 10-hydroxy-9, 10-dihydro-9-oxa-10-phosphaphenanthrene-10-oxide (DOPO-OH) and piperazine to develop transparent flame-retardant epoxy resin (EP) with good mechanical properties. According to the findings, PAHDOPO can be used as a high-performance flame retardant of EP with good mechanical qualities, transparency, and flame retardancy all at the same time [29].

Another study sheds light on the simple manufacturing of ecologically friendly flame retardants and investigates a new method to improve the fire safety and mechanical properties of Epoxy resins (EP) [30].

The compression molding process was utilized to create hybrid composites with varying weight percentages of glass and ramie fibers and stacking sequences. The physical (density, water absorption, and wear resistance) and mechanical (tensile strength, hardness, and impact strength) properties of glass and ramie fibers were investigated, as well as the effect of stacking sequencing. The tensile strength of glass fiber composite stacking (RR to RGR) was lower than that of ramie fiber composite stacking (GG to GRG). The broken composite surface’s microstructure revealed cavities, delamination, fiber interfacial bonding with the matrix, fiber pull-out, and matrix distribution [31].

These studied the performance enhancement of the eco-friendly plaster with additive materials, such as TIO_2_, and so on, other than Epoxy Resin and Epoxy Hardener. Hence, the field is open for studying the impact of Eco-Friendly Plaster Using Epoxy Resin and Epoxy Hardener, especially on Mechanical Properties under Compression and Tension. The cause of this study is due to the good behavior of individual Epoxy. In this regard, this aims to study BISCO plaster (BRP) and a mixture of resin and hardener in three proportions (30%, 45%, and 60%).

## 2. Materials and Methods

### 2.1. The Mixture Materials

- Epoxy resin: It is made up of reactive chemical intermediate with at least two epoxy or hydroxyl groups. They are encouraged to cross-link, which causes polymerization into hard three-dimensional chemical lattices of varying sorts and regularities [32].
- Epoxy hardener: Polyamides and amidoamine epoxy hardeners are made up of aliphatic chains linked together by amide bonds. This distinct backbone gives toughness to the final thermoset, which translates into observed performance improvements in areas such as impact resistance, crack resistance, and substrate adherence [33].
- BSCO plaster is obtained from Bilateral Specialized Company (BSCO) for Industry Saudi Limited Liability Company. It is a cement-based blended powder that can be applied using a trowel placed in typical thicknesses.

### 2.2. Mold Type and Dimensions

For the compression test, we chose cube specimens with dimensions of (50 mm × 50 mm × 50 mm). For the tension test, cylindrical specimens with a length of 100 mm and a diameter of 50 mm were chosen. The specimens for this research were given a standard size by these measurements, allowing for accurate and dependable testing and analysis of the material’s mechanical characteristics. The methodology of this study consists of three parts, the first is selecting the dimensions of samples based on standard (ASTM D638-14 [34] TYPE V) including drawing the specimen dimension on SolidWorks 2023 in order to prepare and coat the samples in the workshop. The second part is conducting a corrosion test by using CS310H and applying the weight loss method. The final part is applying a tensile test by using a universal testing machine (UTM).

### 2.3. Sample Preparation

We had to decide on the proportions of plaster in the mixture, out of the available options: 30%, 45%, and 60%, after selecting the materials and figuring out their shapes. Plaster makes up these percentages; epoxy and epoxy hardener make up the remaining portion.

As we previously mentioned, there will be two shapes for each mixing ratio: five cubic samples and five cylindrical samples. In Table 1, the mixing ratios are displayed.

### 2.4. Mixing Method

First, determine the percentage of plaster, for example, 30%. We bring a bowl, a scale, and a stick for mixing. We put the container on the scale and zero it, then we put on the epoxy resin (1554.8 g)

- In the same way, we put the hardener (777.4 g).
- Mix for five to eight minutes until the mixture changes in consistency (mix slowly to obtain the best result of a bubble-free mixture).
- Add the plaster to the mixture slowly while mixing (1000 g).
- After adding the plaster, we continue mixing until the mixture is homogeneous.
- Pour the mixture into the molds, (Cube/cylinder).
- After at least three days, the samples are collected and prepared for testing.

### 2.5. Compression Test and Tensile Test

#### 2.5.1. Tensile Test

The most frequent deformation technique for polymer testing is tensile testing, which involves clamping a specimen between grips that move apart at a consistent rate. However, utilizing the constant separating speed of the grips to measure sample strain is fraught with inaccuracy because the testing machine will deform under the applied pressure, and slippage of the sample within the grips is not unusual. We use Splitting Tensile Test (ASTM C496) [35] for cylindrical samples, ASTM C496 serves as a crucial guideline in the realm of material testing, specifically addressing the evaluation of the splitting tensile strength of cylindrical concrete specimens. This standardized method, established by ASTM International, involves subjecting a cylindrical concrete sample to a diametral compressive force along its length until the point of failure is reached. The key aspect of this testing procedure is the induction of tensile stresses on the plane containing the applied load, accompanied by relatively high compressive stresses in the region immediately surrounding the load.

Applying pressure from opposite sides to understand how the material responds under tension. What makes this test distinctive is that, despite the compressive force being applied, the concrete tends to fail due to tension rather than compression. This occurrence can be attributed to the areas under load experiencing a state of triaxial compression, allowing them to endure significantly higher compressive stresses compared to what a uniaxial compressive strength test might suggest.

Tensile test findings are used to determine a wide range of specimen properties. Strain is determined by the change in length of the sample’s gauge length G and can be written as engineering strain AG/G or true strain loge (G/Go), where G is the gauge length at a given time and Go is the initial gauge length. The outcomes derived from implementing ASTM C496 [35] play a pivotal role in the engineering and design of structural lightweight concrete members. By gaining insights into the material’s splitting tensile strength, engineers can make informed decisions about its performance under tension, ultimately contributing to the creation of resilient and durable structures. Additionally, these test results are instrumental in determining the development length of reinforcement, offering valuable data for reinforcing concrete structures effectively.

A tensile test is used to determine a material’s yield strength. The test results are shown on a stress–strain curve. The yield strength of a material is the stress at the point where the stress–strain curve deviates from proportionality.

Young’s modulus is the angle at which the slope of the stress strains curve’s beginning (linear part). A tangent modulus (slope of a tangent to the stress–strain curve) or a secant modulus (slope of a line drawn from the origin to a predetermined (typically 2%) strain value on the stress–strain curve) is used to establish a modulus when there is no linear section to the curve in polymers. In principle, the yield stress is the lowest stress that causes permanent deformation when the load is removed [36,37].

In essence, ASTM C496 goes beyond being a testing protocol; it acts as a cornerstone for understanding how concrete behaves under specific conditions, guiding the construction industry in optimizing the design and performance of concrete structures.

#### 2.5.2. Compression Test

The compression test is like giving materials a little workout to see how they handle pressure. To do this, we sandwich the material between two plates and start pushing down on it.

**Figure 1:**
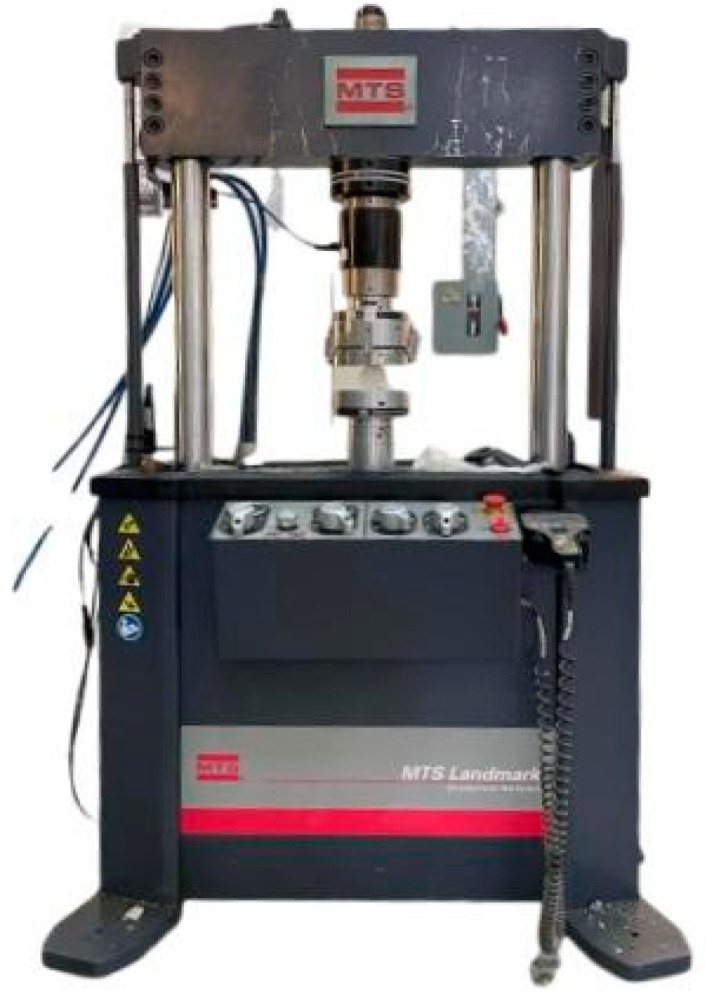
Tensile and compression testing device MTS.

We keep applying more and more pressure until either the material cannot take it anymore, or it reaches its maximum strength. This helps us figure out important things about the material, like how strong it is, how stiff it behaves, and how it deforms under pressure. During the test, we measure the load (the force we are applying) and how much the material squishes or deforms. All these data help us create a stress–strain curve, which is like a roadmap showing how the material handles stress until it eventually gives in. The highest point on this curve is like the material’s superhero moment—it is the maximum compressive strength, showing us the highest stress, its cross-sectional area. In our recent experiment, we took some cube-shaped samples out of their molds after three days. These cubes were then put through the compression test. Taking them out of the molds and the whole curing process went smoothly, no hiccups there. The machine in Figure 1 used is of type MTS-Landmark 810 universal testing machine, Eden Prairie, MN, USA. We placed these cubes in a universal testing machine (UTM), and applied pressure at a steady speed of (1 mm/min). It was like a slow-motion showdown as we watched to see how these cubes handled the pressure until they could not take it anymore according to ASTM D695 [38]. After the test, we crunched the numbers to understand the material’s strength and behavior under all that squeezing. Typically, specimens are squeezed between flat hardened steel plates during compression testing. The stress–strain curve obtained is similar to that of tensile tests, and the same “toe region” and stress and strain criteria apply.

### 2.6. ANSYS Program

**Figure 2:**
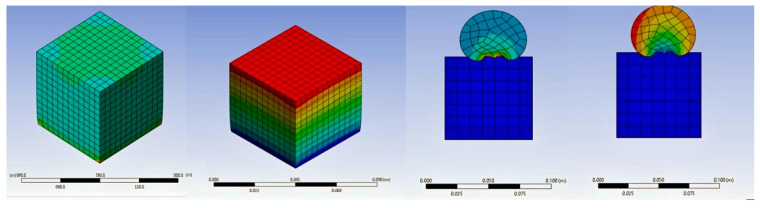
Simulation in ANSYS program.

The utilization of the ANSYS 2023 R1 software program in this study underscores its exceptional capability to optimize a wide range of features, encompassing everything from boundary conditions to the design of geometric elements [39]. ANSYS stands out as an incredibly versatile tool that seamlessly integrates diverse physics to facilitate comprehensive analyses. In the specific context of this research, the primary software employed for simulation-driven design was the ANSYS 2023 R1 Discovery software, which proved to be of utmost importance in unraveling the complexities associated with the behavior of eco-friendly plaster. The initial phase of the investigation focused on establishing boundary conditions through the meticulous execution of dynamic explicit/implicit tensile and compression tests. These tests were meticulously performed on cylindrical and cubic samples, each of which was subjected to varying mixing ratios of 30%, 45%, and 60%. The dynamic nature of the testing procedure facilitated a thorough exploration of the response of the material under different conditions, thereby providing invaluable insights into its mechanical properties. Once the tests were completed, the results were carefully extracted and rigorously compared with those obtained from experimental samples. This comparative analysis played a crucial role in validating the accuracy and reliability of the ANSYS simulation, by juxtaposing the simulated outcomes with real-world experimental data, a deeper understanding of the performance of Mixture under diverse scenarios was achieved. The ANSYS Discovery software emerged as a pivotal tool in this study, seamlessly integrating interactive geometry modeling, high-fidelity simulation, and real-time physics simulation. Its user-friendly interface facilitated a streamlined workflow, enabling efficient analysis and design processes [40,41]. The simulation-driven approach not only expanded the study but also provided a comprehensive platform for evaluating the behavior of Mixture in a variety of scenarios. The integration of simulation into the research methodology allowed for a detailed examination of the material’s response to different conditions, thus enhancing the overall accuracy of the study. The comparison between experimental curves and simulated results provided valuable insights into the predictive capabilities of the ANSYS software, enabling us to draw more precise conclusions about the mechanical characteristics of Mixture. In conclusion, the ANSYS software program Discovery played a pivotal role in this study, providing a comprehensive solution for simulation-driven design. By combining interactive modeling with high-fidelity simulation, a thorough analysis of the behavior of Mixture was facilitated. The ANSYS program employs specific inputs and compares product behavior to physics. The ANSYS software program Discovery was the primary simulation-driven layout tool used in this investigation. A dynamic explicit/implicit approach was used to establish the boundary conditions, with a fixed support at the bottom of the sample and a displacement support at the top of the plaster samples, as illustrated in Figure 2. The integration of simulation into the research methodology not only validated the accuracy of the ANSYS software but also contributed to a more nuanced understanding of the material’s performance in various scenarios, thereby laying the foundation for future advancements in Mixture construction materials (See Figure 2).

### 2.7. Emission Test

**Figure 3:**
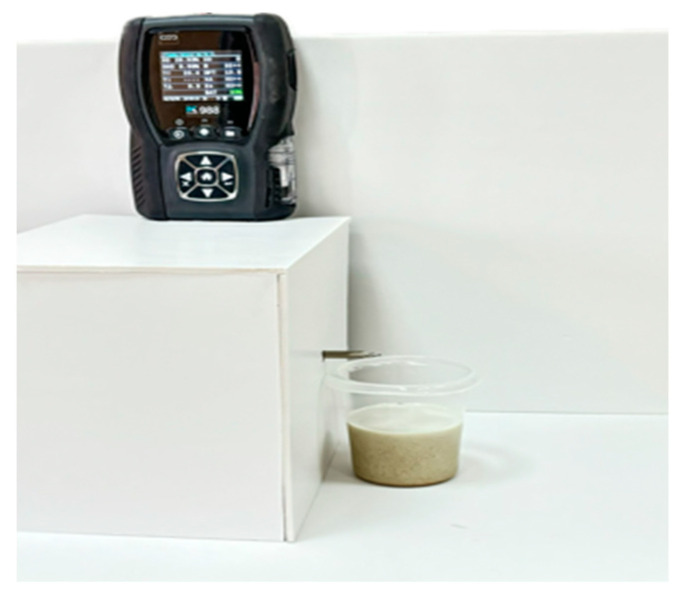
Emission Test setup.

The level of air pollutants emitted is determined using an emission test. The purpose of an emission test is to aid in the reduction of pollutants that are damaging to the environment. An industrial flue gas analyzer is a device as shown in Figure 3 that is employed to quantify the composition of flue gases stemming from industrial combustion procedures. The analyzer has the capacity to quantify a variety of distinct contaminants, including carbon monoxide, nitrogen oxides, sulfur dioxide, and particulate matter. In order to rectify the formation of a small mixture, the thermocouple was temporarily relocated closer to the mixture by a distance of 2–3 cm, and readings were continuously gathered. To ascertain whether there were any emissions of pollutants during the production process or the utilization of eco-friendly plaster, emissions tests were conducted. In this particular investigation, the Kane 988 automotive Diagnostic Exhaust Gas Analyzer manufactured in, Welwyn Garden City UK was the specific type of flue gas analyzer that was employed.

## 3. Results and Discussion

This section of the research endeavors to elucidate and expand upon the comprehensive and intricate mechanical properties of the material under investigation, thereby providing a comprehensive understanding of its behavior under various loads. Moreover, it aims to present the noteworthy and significant findings obtained from a series of meticulously conducted compression and tensile tests, which were meticulously designed and implemented to comprehensively evaluate the material’s response to applied forces. Additionally, this section will delve into a thorough and comprehensive analysis of the results obtained from the emission tests, which were undertaken to ascertain the material’s potential for emitting harmful substances. These aforementioned tests, namely, compression, tensile, and emission tests, were meticulously performed on three different mixing ratios, each ratio includes five test samples and one simulation sample.

### 3.1. Compression Stress Results

**Figure 4:**
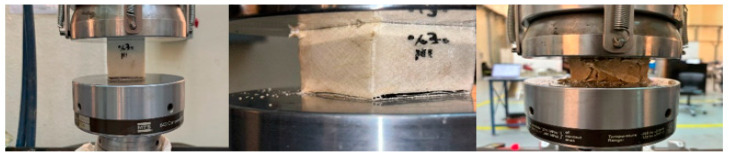
The sample (during) Compression test of BSCO Plaster 30%.

In this part, the properties and effects that appeared during the test are discussed. Figure 4 shows the sample under compression test of BSCO Plaster at 30%.

**Figure 5:**
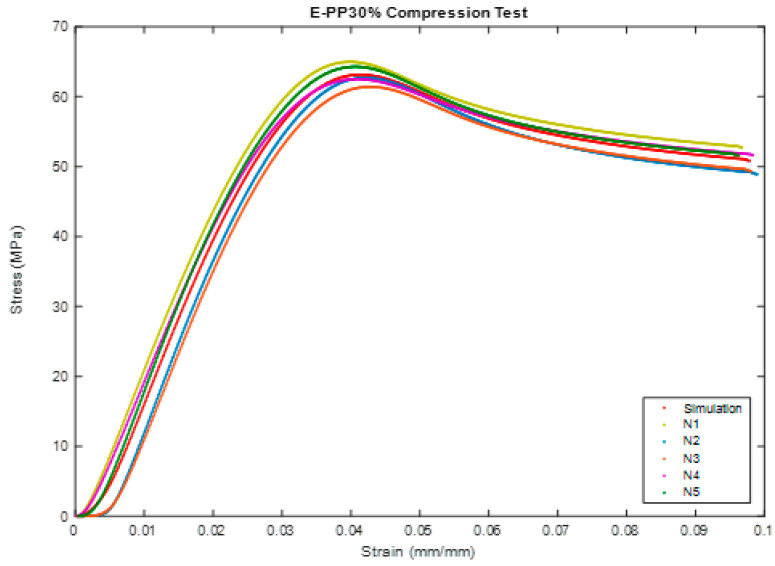
Stress–strain curve in Compression test of BSCO Plaster 30%.

Based on the results of the unconfined compression test, the Compression of BSCO Plaster at 30% for five samples is shown in Figure 5. It presents the strain and stress relation. As shown, the stress increases linearity until it reaches 0.04 mm, then it starts to decrease slightly by increasing in strain. The modulus of elasticity (MPa), UTS (MPa), Yield strength (MPa), Yield strain (mm), Modulus of resilience (kJ/m^3^), and ductility of the samples are tabulated in Table 2.

**Figure 6:**
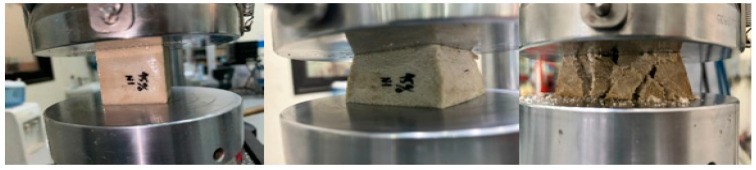
The sample (during) Compression test of BSCO Plaster 45%.

**Figure 7:**
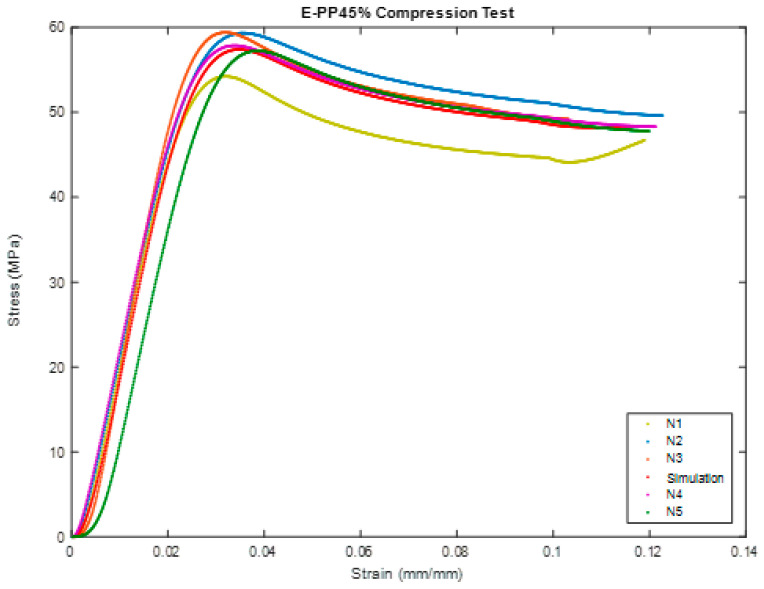
Stress–strain curve in Compression test of BSCO Plaster 45%.

Figure 6 shows the sample during compression test of BSCO Plaster at 45%. Figure 7 depicts the Compression of BSCO Plaster at 45% based on the findings of the unconfined compression test for test samples. It depicts the strain–stress relationship. As seen, the stress grows linearly until it reaches 0.04 mm, at which point it begins to decrease slightly as strain increases. Table 3 lists the modulus of elasticity (MPa), UTS (MPa), yield strength (MPa), yield strain (mm), modulus of resilience (kJ/m^3^), and ductility of the samples.

**Figure 8:**
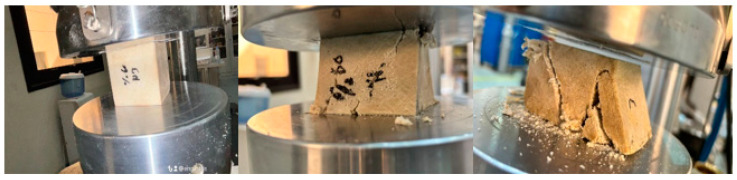
The sample (during) Compression test of BSCO Plaster 60%.

**Figure 9:**
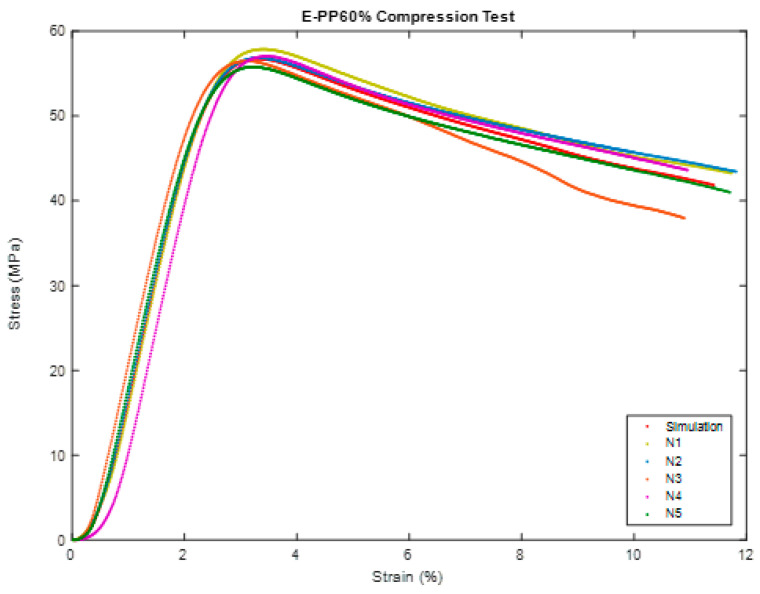
Stress–strain curve in Compression of BSCO Plaster 60%.

Figure 8 and Figure 9 depict the unconfined compression test, the Compression of BSCO Plaster at 60% for five samples. Table 4 lists the modulus of elasticity (MPa), UTS (MPa), yield strength (MPa), yield strain (mm), modulus of resilience (kJ/m^3^), and ductility of the samples.

In this test, our choice of the best mixing ratio will depend on several factors:

In the beginning, we will rely on the simulation results (ideal) because they provide the best environment for testing. They give us the best possible test results that we can obtain. We will compare the different mixing ratios.

#### 3.1.1. Simulation Comparison (Ideal)

This study analyzes the mechanical characteristics of different material compositions, focusing on their modulus of elasticity, ultimate tensile strength (UTS), yield strength, yield strain, modulus of resilience, and ductility. Understanding these properties is crucial for selecting materials suitable for specific applications. The analysis covers compositions of 60%, 30%, and 45% and highlights the advantages and suitability of each composition for different structural requirements.

(a) Modulus of Elasticity (MOE)

The material composition with 60% content exhibits remarkable rigidity, as indicated by its high MOE value of 2969.1 MPa. This high rigidity makes it an ideal choice for load-bearing structures were maintaining structural integrity and minimizing deformations are of utmost importance. Applications such as support frames or elements requiring minimal flexion can benefit from the inherent stiffness of the 60% material.

(b) Ultimate Tensile Strength (UTS)

The material composition with 30% content demonstrates the highest UTS value of 63.2 MPa, indicating its exceptional ability to withstand substantial pulling forces. This composition is well suited for applications requiring strength in tension, such as hanging fixtures or suspended elements.

(c) Yield Strength

The 30% material composition shows a marginal advantage with a slightly higher yield strength of 56.8 MPa. This characteristic is crucial in scenarios where materials are needed to retain their shape under stress. Architectural elements or precision engineering components that require shape and structural integrity can benefit from the 30% material.

(d) Yield Strain

The material composition with 30% content exhibits a slightly elevated yield strain of 0.0305 mm. This suggests that the 30% material can endure a greater amount of deformation before reaching a point of irreversible alterations. It is particularly advantageous in applications requiring materials to withstand repetitive cycles of deformation, such as hinges or joints.

(e) Modulus of Resilience

The material composition with 30% content demonstrates exceptional performance in terms of modulus of resilience, with a value of 0.8681 kJ/m^3^. This characteristic is crucial in situations where the material needs to effectively absorb and dissipate energy. Engineering applications involving dynamic loads or impact resistance can benefit from the energy absorption capabilities of the 30% material.

(f) Ductility

The material composition with 45% content exhibits noteworthy ductility, measuring at a value of 16.69. It is suitable for applications requiring flexibility and the ability to endure bending or stretching forces. The 45% material offers versatility, making it suitable for situations that demand a delicate balance between strength and pliability, such as wall coverings subject to potential bending or distortion.

Accordingly, the mechanical characteristics of different material compositions highlight their suitability for specific applications. The 60% material composition demonstrates high rigidity, making it ideal for load-bearing structures. The 30% composition exhibits exceptional tensile strength, yield strength, yield strain, and resilience, making it suitable for applications involving tension, repetitive deformation, energy absorption, and impact resistance. The 45% composition offers notable ductility and versatility, making it suitable for applications requiring flexibility and a balance between strength and pliability. Understanding these characteristics enables informed material selection for various engineering and construction projects.

#### 3.1.2. Experimental Comparison

But in reality, this comparison is incomplete because it is (ideal), we selected the best sample of each ratio that we tested, “the closest to the simulation results in the curve and the results of the tables”.

After selecting the best samples as shown in Table 5, Table 6 and Table 7, we start comparing the mechanical properties:

- Modulus of Elasticity: The highest modulus of elasticity (Moe) value of 2987.5 MPa is attributed to N5, which is followed closely by N2 at 2585.5 MPa and N4 at 2521.13 MPa.
- Ultimate Tensile Strength (UTS): The UTS of N2 is the highest (62.71 MPa), followed by N4 (57.87 MPa), and then N5 (55.78 MPa).
- Yield Strength: In order of yield strength, N2 has the highest (54.95 MPa), followed by N5 (55.18 MPa), and then N4 (55.23 MPa).
- Yield Strain: In terms of yield strains, N2 has the highest yield (0.0305 mm), followed by N5 (0.0287 mm), and then N4 (0.0268 mm).
- Modulus of Resilience: As a result, N2 has the highest modulus of resilience (0.837 kJ/m^3^), followed by N5 (0.793 kJ/m^3^), and then N4 (0.739 kJ/m^3^).
- Ductility: The highest level of ductility (11.91) is exhibited by N5, followed by N2 (10.94), and subsequently N4 (10.90).
- Stiffness: N5 exhibits the greatest rigidity (the highest Modulus of Elasticity), while N2 is in close proximity.
- Strength: N2 possesses the highest Ultimate Tensile Strength (UTS) and yield strength.
- Deformability: N2 showcases the highest yield strain, signifying superior deformability.
- Energy Absorption: N2 demonstrates the highest resilience modulus.
- Ductility: N5 emerges as the most ductile, with N2 following closely.

### 3.2. Tensile Stress Results

**Figure 10:**
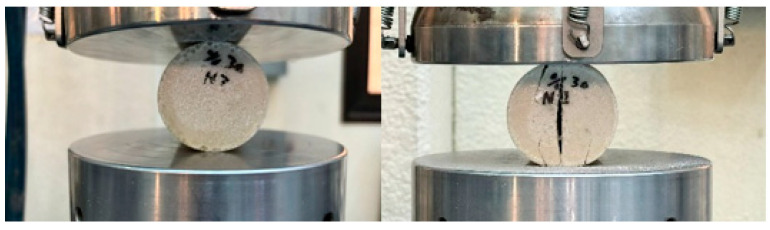
Pictures of the sample (during) Tensile test of BSCO Plaster 30%.

**Figure 11:**
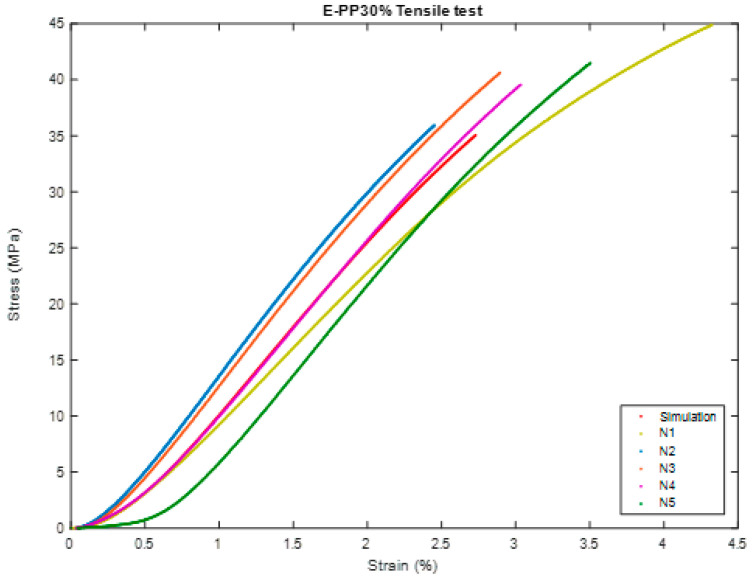
Stress–strain curve in Tensile test of BSCO Plaster 30%.

**Figure 12:**
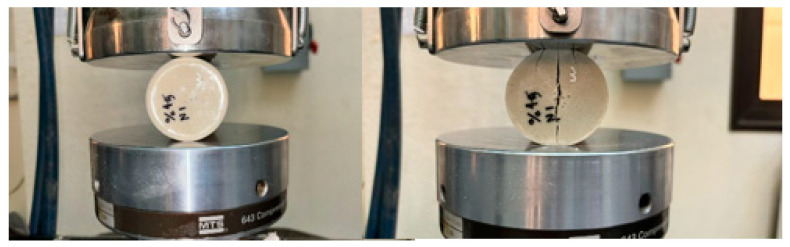
The sample (during) Tensile test of BSCO Plaster 45%.

**Figure 13:**
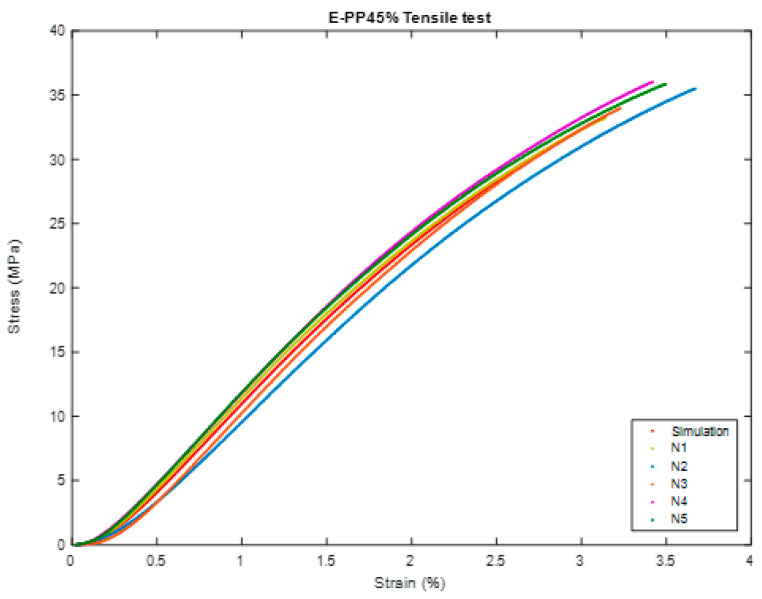
Stress–strain curve in Tensile test of BSCO Plaster 45%.

**Figure 14:**
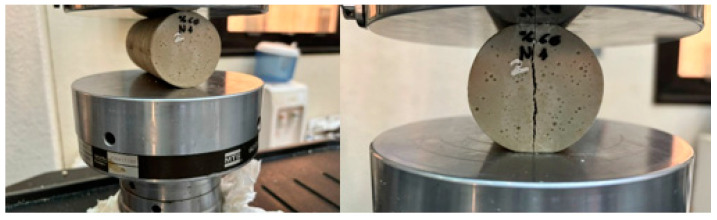
The sample (during) Tensile test of BSCO Plaster 60%.

**Figure 15:**
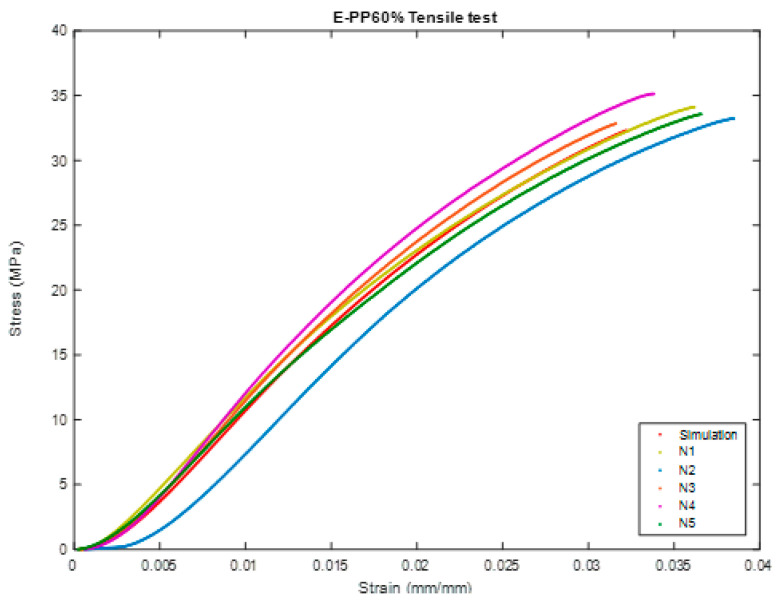
Stress–strain curve in Tensile test of BSCO Plaster 60%.

In this test, we will perform the same comparison steps in the previous test and will do the same in Figure 10, Figure 11, Figure 12, Figure 13, Figure 14 and Figure 15.

#### 3.2.1. Simulation Comparison (Ideal)

As shown in Table 8, Table 9 and Table 10:

- Modulus of Elasticity: 30% has a modulus of elasticity of 1570.27 MPa, indicating high stiffness suitable for applications requiring structural integrity; 45%, with a slightly lower value of 1317.75 MPa, provides a more flexible material, advantageous when elasticity is desired; and 60%, at 1408.46 MPa, strikes a balance between stiffness and flexibility, making it suitable for applications requiring a compromise between the two.
- Ultimate Tensile Strength (UTS): 30% leads with the highest ultimate tensile strength (UTS) at 41.87 MPa, rendering it durable and impervious to fracturing when subjected to tension. The UTS of 45% measures at 34.90 MPa, affording considerable sturdiness. In contrast, 60% possess a UTS of 33.78 MPa, a strength that is comparable to that of 45%. This composition is well suited for applications where an exceptionally elevated tensile strength is not the principal requirement.
- Yield Strength: 30% demonstrates exceptional performance in terms of yield strength, measuring at 39.12 MPa. This attribute is particularly vital in situations that necessitate precise control over deformation. On the other hand, 45% exhibits a yield strength of 29.81 MPa, showcasing commendable resistance to plastic deformation. This characteristic renders it appropriate for applications that require a delicate balance between strength and deformation. Lastly, 60% boasts a yield strength of 28.25 MPa, providing ample robustness for applications that deem moderate yield strength acceptable.
- Yield Strain: 30% exhibit a yield strain of 0.0293 mm, which provides a moderate capacity for deformation prior to plastic deformation; 45%, possessing a yield strain slightly lower at 0.0268 mm, presents an advantage in scenarios where minimal deformation is preferred; and 60%, showcasing a yield strain of 0.0263 mm, achieves a compromise between the deformation capabilities of 30% and 45%.
- Modulus of Resilience: 30% demonstrates superior performance in terms of modulus of resilience at 0.5788 kJ/m^3^, which is of utmost importance in applications that necessitate the ability to absorb and recover elastic energy. At 45%, possessing a value of 0.4002 kJ/m^3^, it showcases commendable energy absorption capabilities, rendering it suitable for applications that require a balance between elasticity and energy absorption. With a value of 0.3727 kJ/m^3^, 60% offers reasonable energy absorption while still maintaining flexibility, thus making it appropriate for applications that demand controlled energy dissipation.
- Ductility: 30%, possessing a ductility value of 3.85, presents a moderate level of ductility that is well suited for the purpose of controlled deformation. With a slightly elevated value of 3.98, 45% is deemed appropriate for applications wherein a certain degree of deformation is considered acceptable. The leading position in terms of ductility is held by 60%, which boasts a value of 4.26, rendering it an exceptional choice for applications necessitating a significant capacity for deformation without experiencing failure.

#### 3.2.2. Experimental Comparison

However, the comparison is incomplete due to the difficulty of providing an integrated environment in the program. Therefore, we chose the best sample for each ratio that closely matched the simulation results as shown in Table 11, Table 12 and Table 13.

- Modulus of elasticity is an indicator of a material’s stiffness and its resistance to deformation when subjected to an applied load. Among the compared materials, N4 demonstrates the highest modulus at 1671.74 MPa, which signifies its exceptional stiffness. N5 closely follows with a modulus of 1357.72 MPa, while N1 lags behind with a modulus of 1317.12 MPa. When designing structures, engineers frequently take into account the modulus of elasticity to ensure specific stiffness requirements.
- Ultimate Tensile Strength (UTS): Ultimate Tensile Strength (UTS) represents the maximum stress a material can withstand before experiencing failure. N4 boasts the highest UTS at 38.03 MPa, indicating its superior strength. N1 and N5 exhibit UTS values of 35.84 MPa and 34.11 MPa, respectively. The choice among these samples may be influenced by the desired strength characteristics, depending on the application.
- Yield strength: Yield strength marks the point at which a material undergoes plastic deformation. N4 demonstrates the highest yield strength at 36.08 MPa, followed by N1 with a value of 28.22 MPa, and N5 with a value of 26.80 MPa. The determination of yield strength is crucial in applications where controlled deformation plays a vital role, such as in the manufacturing of components subjected to repeated loading.
- Yield Strain: Yield Strain: The measurement of yield strain entails the evaluation of the extent of deformation that a material can withstand prior to experiencing plastic deformation. Among the samples tested, N4 exhibits the lowest yield strain at a value of 0.0174 mm, while N1 and N5 demonstrate slightly higher values at 0.02422 mm and 0.0243 mm, respectively. The selection of these samples is contingent upon the acceptable degree of plastic deformation in a given application.
- Modulus of Resilience: The assessment of the modulus of resilience serves to gauge a material’s capacity to absorb energy before irreversible deformation occurs. N4, with a resilience value of 0.3145 kJ/m^3^, manifests the lowest resilience, implying a greater likelihood of experiencing plastic deformation. Comparatively, N1 (0.3417 kJ/m^3^) and N5 (0.3258 kJ/m^3^) exhibit slightly higher values for resilience. This particular property assumes paramount significance in applications where energy absorption constitutes a pivotal consideration.
- Ductility: Ductility signifies a material’s ability to undergo substantial plastic deformation prior to rupture. Among the specimens tested, N1 showcases the highest level of ductility at 4.23, followed by N5 (4.14) and N4 (2.95). Depending on the intended application, a material characterized by superior ductility may be favored in situations where deformation preceding failure assumes critical importance.

### 3.3. Emission Test

As we know, our research aims to reduce emissions into the environment, and to make sure we are on the right track, we must conduct an emission test, which is performed every 5 min to 30 min. The test site was arranged to ensure stability of parameters and heights. The gases tested were carbon dioxide, sulfur dioxide, and nitrogen dioxide. It was performed in three ratios: 30%, 45%, and 60% as shown in Table 14.

Carbon Dioxide (CO_2_): The results for “carbon dioxide (CO_2_) are zero”, as the table shows. This indicates the absence of carbon dioxide (CO_2_) in all proportions tested. Regarding the environment, climate, and human health, there are many impacts. Although carbon dioxide is a natural part of the Earth’s atmosphere and essential for life, increases in concentrations associated with human activity have harmful effects. Carbon dioxide is one of the primary greenhouse gases causing global warming.

Sulfur Dioxide (SO_2_): As shown in the table, sulfur dioxide (SO_2_) appeared in the test at different times. At 30%, it appeared between 10 and 20 min, and the percentage was the highest. At 45%, it appeared 5 min early and lasted 15 min, while at 60%, it appeared 10 to 25 min late. Reducing sulfur dioxide emissions contributes to reducing acid rain and improving air quality in general. One of the main air pollutants that might lead to poor air quality is SO_2_, which is emitted into the atmosphere. It can cause the production of fine particulate matter (PM) and aggravate respiratory problems, especially in people who already have bronchitis or asthma.

Nitrogen Dioxide (NO_2_): As is clear, nitrogen dioxide appeared at a rate of 30% between 15 and 20 min. However, it does not exist at 45% and 60%, and this is a good indicator to avoid its negative effects. Long-term exposure to nitrogen dioxide has been associated with heart problems, such as a higher risk of heart attacks and other heart diseases. The heart and blood vessels may be affected by the inflammatory reaction caused by nitrogen dioxide.

## 4. Conclusions

In this research, BSCO Plaster (BRP) was studied and combined with varying percentages of solids and resins (30%, 45%, and 60%). In order to choose the optimal ratio, economically and environmentally, the research’s goal was to conduct experiments and verify the mechanical properties of different ratios. Of course, protecting the environment is essential for the future of the world, and for Saudi Arabia’s 2030 vision to achieve zero carbon neutrality by 2060. In the near future, the development of BSCO Plaster (BRP) may help achieve this goal. ANSYS is used in this research, a simulation technology, to assist us in our research by displaying simulation results. The test results were plotted using MATLABR2023b. We compared the simulation results with specimens subjected to compression and tensile testing. By combining BSCO Plaster (BRP), epoxy resin, and epoxy hardener, we were also able to identify gases released during emissions testing. The emission test also did not reveal the presence of carbon dioxide in any quantity, which is a positive indicator. However, sulfur dioxide and nitrogen dioxide were detected, and the highest emission rate was 30%. The lowest emission rate was 60%. As we have noted, a material’s level of environmental friendliness cannot be determined by its CO_2_ content alone. Being green requires thorough testing and knowledge of all emissions, and after concluding the process of experimentation and conducting a detailed analysis of the specimens, a more lucid understanding was obtained. Consequently, we are now able to designate the optimal blending proportion that can furnish us with the most superior structural characteristics of the plaster utilized for wall cover, In the thorough examination of materials for the purpose of covering walls, the choice of a 30% mixing ratio emerges as the most advantageous option. This recommendation is based on a comprehensive analysis of key mechanical properties, taking into account both tensile and compressive stress scenarios.

The property of stiffness and elasticity, exemplified by N2 with a modulus of elasticity (Moe) of 2585.5 MPa, signifies the delicate balance between rigidity and flexibility. This particular attribute is crucial for wall coverings in order to maintain their structural integrity and resist deformation under various loads. The strength and durability of N2, demonstrated by its Ultimate Tensile Strength (UTS) of 62.71 MPa, positions it as the top choice, surpassing both N4 and N5. This characteristic is essential for wall coverings that are expected to endure a range of stresses, ensuring their longevity and reliability. The deformability and adaptability of N2, highlighted by its superior yield strain of 0.0305 mm, enable controlled plastic deformation. This property plays a significant role in ensuring adaptability to changing conditions and potential impacts. The energy absorption and resilience of N2, evidenced by its highest modulus of resilience of 0.837 kJ/m^3^, accentuate its ability to absorb and dissipate energy before reaching a state of irreversible deformation. In wall-covering applications, where resilience is crucial for withstanding impacts or dynamic forces, N2 with a 30% mixing ratio is an optimal choice. While N5 emerges as the most ductile, N2 still maintains a commendable level of ductility with a rating of 10.9425. Ductility is crucial in scenarios where materials need to undergo significant plastic deformation before reaching rupture. The 30% mixing ratio strategically balances ductility with other essential properties, ensuring a material that is flexible yet resilient. The synthesis of these mechanical properties establishes the 30% mixing ratio with N2 as an exemplary choice for wall-covering applications.

Its ability to seamlessly integrate high stiffness, superior strength, controlled deformability, energy absorption, and commendable ductility provides a foundation for optimal performance. In conclusion, while the 30% mixing ratio with N2 is the frontrunner, a thoughtful final decision must consider the specific demands of the research. Customizing the selection to precisely align with the requirements of the application will guarantee an optimal balance of mechanical attributes, meeting both performance criteria and professional standards.

## Figures and Tables

**Table 1:** Mixing Ratios.

Percent%	30%	45%	60%
Epoxy resin	1554.8 g	1356 g	986 g
Epoxy hardener	777.4 g	680 g	493 g
BSCO Plaster	1000 g	1664 g	2220 g
Cube/cylinder	6/6	6/6	6/6

**Table 2:** Mechanical properties in Compression of BSCO Plaster 30%.

30%	Modulus of Elasticity (MPa)	UTS (MPa)	Yield Strength (MPa)	Yield Strain (mm)	Modulus of Resilience (kJ/m^3^)	Ductility
Simulation	2474.3	63.18144	56.82131	0.030515343	0.868099727	10.845816
N1 (sample 1)	2404.9	64.98655	61.46703	0.03200549	0.983641207	10.77472
N2 (sample 2)	2585.5	62.71313	54.94956	0.030479743	0.837424229	10.9425
N3 (sample 3)	2484.6	61.4094	56.56342	0.033143333	0.937350142	10.9013
N4 (sample 4)	2340.8	62.50931	55.16697	0.028548981	0.787480388	10.73028
N5 (sample 5)	2555.8	64.2888	55.95957	0.02839917	0.794602671	10.88028

**Table 3:** Mechanical properties in Compression of BSCO Plaster 45%.

45%	Modulus of Elasticity (MPa)	UTS (MPa)	Yield Strength (MPa)	Yield Strain (mm)	Modulus of Resilience (kJ/m^3^)	Ductility
Simulation	2592.374532	57.619254	55.79183	0.02896498	0.80932419	16.694224
N1	2547.173436	54.23194	53.05866	0.0275384	0.73057524	27.37466
N2	2535.588231	59.33504	55.15913	0.02635071	0.72674109	15.02792
N3	3017.690321	59.41383	59.23162	0.0301048	0.89157804	15.11488
N4	2521.130235	57.86549	55.22867	0.02677722	0.7394352	10.9013
N5	2340.290439	57.24997	56.28107	0.03405377	0.9582914	15.05236

**Table 4:** Mechanical properties in Compression of BSCO Plaster 60%.

60%	Modulus of Elasticity (MPa)	UTS (MPa)	Yield Strength (MPa)	Yield Strain (mm)	Modulus of Resilience (kJ/m^3^)	Ductility
Simulation	2969.14798	56.799008	55.527024	0.02864182	0.79590719	11.601648
N1	2983.29552	57.87134	55.48568	0.0272424	0.75578154	11.92556
N2	2978.13767	56.84723	56.83703	0.0324285	0.92156981	12.0089
N3	2988.59644	56.47208	54.78936	0.0256506	0.70268998	11.07346
N4	2908.17811	57.0289	55.34203	0.0291439	0.80644129	11.08688
N5	2987.53215	55.77549	55.18102	0.0287437	0.79305334	11.91344

**Table 5:** Comparison between Simulation and Closest Sample of BSCO Plaster 30% in comparison.

Sample 30%	
Simulation	Closest Sample: N2
Modulus of Elasticity: 2474.3 MPa	Modulus of Elasticity: 2585.5 MPa (Closest)
UTS: 63.18144 MPa	UTS: 62.71313 MPa
Yield Strength: 56.82131 MPa	Yield Strength: 54.94956 MPa
Yield Strain: 0.030515343 mm	Yield Strain: 0.030479743 mm
Modulus of Resilience: 0.868099727 kJ/m^3^	Modulus of Resilience: 0.837424229 kJ/m^3^
Ductility: 10.845816	Ductility: 10.9425

**Table 6:** Comparison between Simulation and Closest Sample of BSCO Plaster 45% in comparison.

Sample 45%	
Simulation	Closest Sample: N4
Modulus of Elasticity: 2592.374532 MPa	Modulus of Elasticity: 2521.130235 MPa (Closest)
UTS: 57.619254 MPa	UTS: 57.86549 MPa (Closest)
Yield Strength: 55.79183 MPa	Yield Strength: 55.22867 MPa (Closest)
Yield Strain: 0.02896498 mm	Yield Strain: 0.02677722 mm
Modulus of Resilience: 0.80932419 kJ/m^3^	Modulus of Resilience: 0.7394352 kJ/m^3^
Ductility: 16.694224	Ductility: 10.9013

**Table 7:** Comparison between Simulation and Closest Sample of BSCO Plaster 60% in comparison.

Sample 60%	
Simulation	Closest Sample: N5
Modulus of Elasticity: 2969.14798 MPa	Modulus of Elasticity: 2987.53215 MPa (Closest)
UTS: 56.799008 MPa	UTS: 55.77549 MPa (Closest)
Yield Strength: 55.527024 MPa	Yield Strength: 55.18102 MPa (Closest)
Yield Strain: 0.02864182 mm	Yield Strain: 0.0287437 mm (Closest)
Modulus of Resilience: 0.79590719 kJ/m^3^	Modulus of Resilience: 0.79305334 kJ/m^3^ (Closest)
Ductility: 11.601648	Ductility: 11.91344 (Closest)

**Table 8:** Mechanical properties in Tensile of BSCO Plaster 30%.

30%	Modulus of Elasticity (MPa)	UTS (MPa)	Yield Strength (MPa)	Yield Strain (mm)	Modulus of Resilience (kJ/m^3^)	Ductility
Simulation	1570.26818	41.870024	39.121478	0.02929044	0.57881219	3.846848
N1	1555.8123	42.96246	40.86177	0.0344317	0.7034701	4.1392
N2	1569.5625	41.41372	38.79734	0.0296515	0.57519966	3.73872
N3	1689.96557	42.05036	39.04433	0.0275864	0.53854625	3.52018
N4	1671.73736	38.03367	36.0832	0.0174303	0.3144705	2.94584
N5	1364.26319	44.88991	40.82075	0.0373523	0.76237445	4.8903

**Table 9:** Mechanical properties in Tensile of BSCO Plaster 45%.

45%	Modulus of Elasticity (MPa)	UTS (MPa)	Yield Strength (MPa)	Yield Strain (mm)	Modulus of Resilience (kJ/m^3^)	Ductility
Simulation	1317.752461	34.90459	29.811234	0.02678684	0.40018788	3.979388
N1	1317.126433	35.84041	28.22042	0.0242206	0.34175775	4.23418
N2	1344.063488	36.01811	30.54695	0.0265856	0.4060545	3.99746
N3	1341.289719	33.97304	30.38082	0.0275872	0.41906088	3.77892
N4	1273.634222	35.50376	30.97855	0.0299489	0.46388675	4.30616
N5	1312.648443	33.18763	28.92943	0.0255919	0.37017954	3.58022

**Table 10:** Mechanical properties in Tensile of BSCO Plaster 60%.

60%	Modulus of Elasticity (MPa)	UTS (MPa)	Yield Strength (MPa)	Yield Strain (mm)	Modulus of Resilience (kJ/m^3^)	Ductility
Simulation	1408.458481	33.775874	28.251228	0.0262996	0.3727238	4.255328
N1	1484.136569	32.84509	29.24351	0.02616376	0.38256013	3.86566
N2	1332.307562	33.56225	27.11476	0.025733	0.34887206	4.37154
N3	1559.093593	35.1347	28.21384	0.02361644	0.33315527	4.09556
N4	1309.036865	33.22539	29.88413	0.03166956	0.47320857	4.79906
N5	1357.717816	34.11194	26.7999	0.02431524	0.32582297	4.14482

**Table 11:** Comparison between Simulation and Closest Sample of BSCO Plaster 30% in Tensile.

Sample 30%	
Simulation	Closest Sample: N4
Modulus of Elasticity: 1570.27 MPa	Modulus of Elasticity: 1671.74 MPa
UTS: 41.87 MPa	UTS: 38.03 MPa
Yield Strength: 39.12 MPa	Yield Strength: 36.08 MPa
Yield Strain: 0.0293 mm	Yield Strain: 0.0174 mm
Modulus of Resilience: 0.5788 kJ/m^3^	Modulus of Resilience: 0.3145 kJ/m^3^
Ductility: 3.85	Ductility: 2.95

**Table 12:** Comparison between Simulation and Closest Sample of BSCO Plaster 45% in Tensile.

Sample 45%	
Simulation	Closest Sample: N1
Modulus of Elasticity: 1317.75 MPa	Modulus of Elasticity:1317.12 MPa
UTS: 34.90 MPa	UTS: 35.84 MPa
Yield Strength: 29.81 MPa	Yield Strength: 28.22 MPa
Yield Strain: 0.0268 mm	Yield Strain: 0.02422 mm
Modulus of Resilience: 0.4002 kJ/m^3^	Modulus of Resilience: 0.3417 kJ/m^3^
Ductility: 3.98	Ductility: 4.23

**Table 13:** Comparison between Simulation and Closest Sample of BSCO Plaster 60% in Tensile.

Sample 60%	
Simulation	Closest Sample: N5
Modulus of Elasticity: 1408.46 MPa	Modulus of Elasticity: 1357.72 MPa
UTS: 33.78 MPa	UTS: 34.11 MPa
Yield Strength: 28.25 MPa	Yield Strength: 26.80 MPa
Yield Strain: 0.0263 mm	Yield Strain: 0.0243 mm
Modulus of Resilience: 0.3727 kJ/m^3^	Modulus of Resilience: 0.3258 kJ/m^3^
Ductility: 4.26	Ductility: 4.14

**Table 14:** Emission test comparison.

Time (min)	30%			45%			60%		
	CO_2_ (ppm)	SO_2_ (ppm)	NO_2_ (ppm)	CO_2_ (ppm)	SO_2_ (ppm)	NO_2_ (ppm)	CO_2_ (ppm)	SO_2_ (ppm)	NO_2_ (ppm)
0	0	0	0	0	0	0	0	0	0
5	0	0	0	0	1	0	0	0	0
10	0	1	0	0	1	0	0	0	0
15	0	2	1	0	1	0	0	1	0
20	0	2	1	0	0	0	0	1	0
25	0	0	0	0	0	0	0	1	0
30	0	0	0	0	0	0	0	0	0

## Data Availability

The datasets used during the current study are available from the corresponding author on reasonable request (due to privacy).
